# Atrial Arrhythmia in Ageing Spontaneously Hypertensive Rats: Unraveling the Substrate in Hypertension and Ageing

**DOI:** 10.1371/journal.pone.0072416

**Published:** 2013-08-27

**Authors:** Dennis H. Lau, Nicholas J. Shipp, Darren J. Kelly, Shivshankar Thanigaimani, Melissa Neo, Pawel Kuklik, Han S. Lim, Yuan Zhang, Karen Drury, Christopher X. Wong, Nicholas H. Chia, Anthony G. Brooks, Hany Dimitri, David A. Saint, Lindsay Brown, Prashanthan Sanders

**Affiliations:** 1 Centre for Heart Rhythm Disorders (CHRD), University of Adelaide and Royal Adelaide Hospital, Adelaide, Australia; 2 Department of Medicine, St. Vincent’s Hospital, University of Melbourne, Melbourne, Australia; 3 University of Southern Queensland, Toowoomba, Australia; Brigham & Women’s Hospital - Harvard Medical School, United States of America

## Abstract

**Background:**

Both ageing and hypertension are known risk factors for atrial fibrillation (AF) although the pathophysiological contribution or interaction of the individual factors remains poorly understood. Here we aim to delineate the arrhythmogenic atrial substrate in mature spontaneously hypertensive rats (SHR).

**Methods:**

SHR were studied at 12 and 15 months of age (n = 8 per group) together with equal numbers of age-matched normotensive Wistar-Kyoto control rats (WKY). Electrophysiologic study was performed on superfused isolated right and left atrial preparations using a custom built high-density multiple-electrode array to determine effective refractory periods (ERP), atrial conduction and atrial arrhythmia inducibility. Tissue specimens were harvested for structural analysis.

**Results:**

Compared to WKY controls, the SHR demonstrated: Higher systolic blood pressure (p<0.0001), bi-atrial enlargement (p<0.05), bi-ventricular hypertrophy (p<0.05), lower atrial ERP (p = 0.008), increased atrial conduction heterogeneity (p = 0.001) and increased atrial interstitial fibrosis (p = 0.006) & CD68-positive macrophages infiltration (p<0.0001). These changes resulted in higher atrial arrhythmia inducibility (p = 0.01) and longer induced AF episodes (p = 0.02) in 15-month old SHR. Ageing contributed to incremental bi-atrial hypertrophy (p<0.01) and atrial conduction heterogeneity (p<0.01) without affecting atrial ERP, fibrosis and arrhythmia inducibility. The limited effect of ageing on the atrial substrate may be secondary to the reduction in CD68-positive macrophages.

**Conclusions:**

Significant atrial electrical and structural remodeling is evident in the ageing spontaneously hypertensive rat atria. Concomitant hypertension appears to play a greater pathophysiological role than ageing despite their compounding effect on the atrial substrate. Inflammation is pathophysiologically linked to the pro-fibrotic changes in the hypertensive atria.

## Introduction

Both ageing and hypertension are known risk factors for atrial fibrillation (AF). Epidemiological studies have established that the odds ratio for developing AF was 2.1–2.2 and 1.4–1.5 for each decade of advancing age and hypertension respectively. [1] While the presence of multiple risk factors is likely to increase the susceptibility and persistence of AF, the pathophysiological contribution or interaction of the individual factors remains poorly understood. [2], [3] Several studies had characterized the substrate for AF in hypertension or ageing individually, but the compounding effect of ageing and hypertension on the atria has not been studied in detail.

The spontaneously hypertensive rat (SHR) is most commonly used in high blood pressure research to represent established human hypertension, with the Wistar Kyoto rat (WKY) as the normotensive control. [4] However, due to the difficulty in performing detailed high density direct contact electrophysiological mapping study in rat atria and the long-held belief that small atria do not fibrillate according to the “critical mass” hypothesis, limited high density atrial mapping data is available from the SHR model [5]–[8]. Recently, we reported on the feasibility of high density multiple-electrode array (MEA) contact mapping studies of superfused small animal atria. [9] Here, we aimed to comprehensively evaluate the atrial electrical and structural remodeling predisposing to AF in ageing SHR with established hypertension using a custom-built high density MEA system and structural analysis.

## Methods

This study was approved by the Animal Ethics Committee of the University of Adelaide, Adelaide, Australia (Approval Number S-078-07). All animal experimentations conformed to the ‘Guide for the Care and Use of Laboratory Animals’ as adopted by National Institutes of Health, USA and all efforts were made to minimize suffering. A total of 32 male rats were included in this study: 16 were SHR and 16 were normotensive WKY control rats at 12 and 15 months of age (n = 8 per time point). The SHR (lifespan 18–30 months; 50% of this colony survive for 21 months) develops hypertension from 2–3 months of age which peaks from 6 months of age onwards with increasing compensatory concentric cardiac hypertrophy, before the onset of heart failure at the age of 15–18 months. [4], [10], [11] The time-points of 12 and 15 months were chosen to allow characterization of the atrial substrate due to established hypertension with maximal left ventricular hypertrophy in mature rats, based on the well-characterized blood pressure and cardiovascular profile of the SHR model prior to the development of heart failure. [11].

All animals were given *ad libitum* access to food and water and were housed in temperature-controlled rooms with 12 h light/dark cycle. For experimentations, they were first anesthetized with intra-peritoneal injection of ketamine (60 mg/kg) and medetomidine (0.5 mg/kg). Systolic blood pressure was then measured non-invasively using an inflatable tail-cuff and pressure transducer in conjunction with a PowerLab system (MLT125/R cuff/transducer and ML125 NIBP controller, ADInstruments, New South Wales, Australia). At least three measurements were performed and the average taken. Transthoracic echocardiography (Vivid 7 with 10 MHz transducer, GE Healthcare, Buckinghamshire, United Kingdom) was used to determine left ventricular wall thickness and systolic fractional shortening from M-mode measurements at the level of the papillary muscles with atria dimensions measured from the apical 4-chamber view. [12] The heart was removed via a midline thoracotomy incision and placed in ice-cold modified HEPES buffer containing in mM (134 NaCl, 4 KCl, 1.2 NaH_2_PO_4_, 1.2 MgSO_4_, 11 glucose and 10 HEPES-NaOH). Both right and left atria were then carefully dissected from the heart for immediate electrophysiological studies.

### Electrophysiological Study

Detailed information about the MEA system setup (Nucleus Medical, Adelaide, Australia) has been described previously. [9] In brief, the MEA measures 4×4.5 mm and consists of 90 silver chloride electrodes of 0.1 mm diameter with 0.5 mm inter-electrode distance. The atrial tissue was placed over the MEA and superfused with modified HEPES solution (pH 7.35) and bubbled with 100% oxygen. 1 mM of 2,3-butanedione monoxime was added to suppress contraction artifacts together with 1.5 mM of CaCl_2_. The superfusate was circulated in a closed loop using a peristaltic pump (Minipuls 3, Gilson Inc., Middleton, USA) and its temperature regulated at 37.5°C with a heater. A lightweight mesh was placed over the tissue to ensure constant tissue contact with the underlying electrodes. The MEA was connected to a computerized recording system (LabSystem Pro, Bard Electrophysiology, Lowell, USA) with a sampling rate of 4 kHz and filtering from 10–500 Hz. For consistency, the atrial tissues were placed over the MEA in the same cranial-caudal and medial-lateral orientation with the epicardial surface in contact with the electrodes.

#### Atrial effective refractory period

Atrial effective refractory period (ERP) was measured at twice diastolic threshold from two sites in each atrium – RA free wall (RAFW), RA appendage (RAA), LA free wall (LAFW) and LA appendage (LAA). This was performed with eight basic (S1) stimuli at 400, 300, 200 and 100 ms followed by a premature (S2) stimulus in 5 ms decrement. Refractoriness was defined as the longest S1–S2 interval not resulting in a propagated response. A total of three ERP measurements were taken from each site at each cycle length and the total averaged. The coefficient of ERP variation (standard deviation/mean×100%) was determined to assess ERP heterogeneity.

#### Atrial conduction

Conduction was assessed during stable S1 pacing at 400, 300, 200 and 100 ms from 2 pre-specified opposing corners of the MEA. Activation maps were created using semi-automated custom-made plaque analysis software (Nucleus Medical, Adelaide, Australia). Each annotation was manually verified with the local activation time annotated to the peak of the largest amplitude deflection on bipolar electrograms. Conduction velocity was calculated from local vectors within each triangle of electrodes to derive a mean conduction velocity for each activation map. [13].

#### Conduction heterogeneity

Conduction heterogeneity was assessed using an established phase mapping method during S1 pacing. [14] In brief, the largest activation time difference between every four adjacent electrodes was first determined. This was then used to create a phase map, with values also displayed as a histogram. Absolute conduction phase delay was taken as the difference between the 5th from the 95th percentile of the phase distribution (P5–95). The conduction heterogeneity index was then obtained by dividing the absolute phase delay by the median (P50).

#### Arrhythmia inducibility and duration

This was assessed with rapid decremental pacing at the atrial appendage, starting from 100 ms cycle length, which was decreased in 5 ms intervals every 2 seconds until either atrial tachycardia/fibrillation (AT/AF) was initiated or there was loss of 1∶1 capture. This protocol of induction was implemented 10 times in total for determining inducibility and duration of induced arrhythmic episodes. AT/AF was defined as rapid regular/irregular atrial activation lasting more than 6 beats. A normalized voltage algorithm was implemented to visualize the wavefront propagation sequence during atrial arrhythmia (Refer to online supplement methods S1).

### Structural Analysis

Following electrophysiological studies, the atrial tissues were immersed fixed with 10% neutralized buffered formalin and paraffin embedded for subsequent light microscopic evaluation. To assess the morphology of atrial myocytes, tissue sections were stained with haematoxylin and eosin (H&E) stain. Measurement of mean myocyte cross-sectional area was performed as previously described. [15] In brief, only myocytes with intact cellular membranes from fields with circular capillary profiles and myofiber shapes were assessed. In total, the circumferences of 40 to 50 cells per atrium were traced digitally to calculate mean cross-sectional area. Sections were stained with picrosirius red to demonstrate the extracellular matrix in a masked protocol. Twenty random images from each atrium of every animal were digitally scanned by Aperio Scanscope (Model CS and ImageScope, Aperio Technologies, Vista, USA) and the accumulation of matrix was quantitated by using a modification of the technique described by Lehr et al in a blinded manner. [16] Immunostaining was also performed with mouse anti-rat CD68 monoclonal antibody (AbD Serotec, Raleigh, USA) for quantification of interstitial macrophage infiltration. Macrophage number in the interstitium of the heart was estimated by counting the number of macrophages in 10 fields under light microscopy with a magnification ×200 per animal from each group and expressed as numbers per section.

### Statistical Analysis

**Figure 1:**
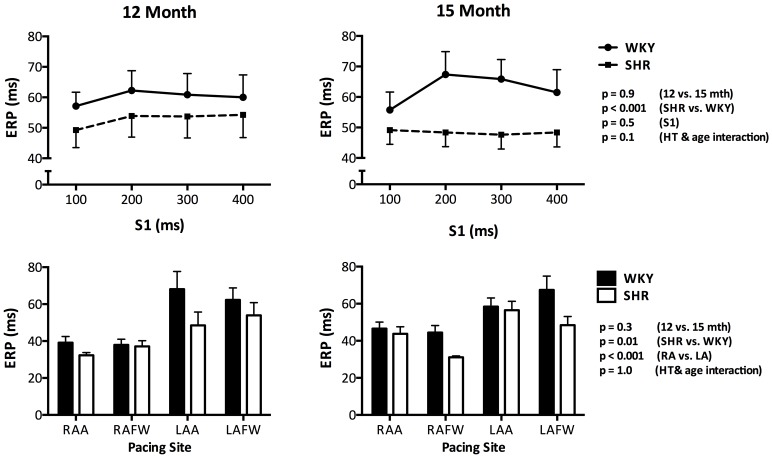
Atrial Refractoriness. Atrial ERP was significantly lower in the SHR as compared to the WKY controls at both time points (Mean ERP from LAFW shown in the top row). Although ERP was not affected by ageing or pacing cycle length (S1), higher ERP was seen in the LA as compared to the RA (Mean ERP with S1 @200 ms shown in the bottom row). Error bars denote standard error of the mean.

All continuous variables are reported as mean ± SD and assessed for normality using the Shapiro-Wilk test. To improve presentation clarity, mean ± standard error of the mean was utilized in Figure 1. Data were compared using the Student’s t-test or Wilcoxon rank sum test according to their distribution. Categorical data was tested using Fisher’s exact statistics. A linear mixed effects model was used to compare changes over time of electrophysiologic data between the two groups, with hypertension, age and their interaction modeled as fixed effects and individual animal fitted as random effect. Statistical significance was established at *P*<0.05.

## Results

Detailed animal characteristics are shown in Table 1. There was no significant difference in body weight between the SHR and WKY controls at both time-points. In these mature adult rats, systolic blood pressure was significantly higher in the SHR as compared to the WKY controls. In particular, systolic blood pressure was found to be stable between 12 and 15 months of age.

**Table 1:** Animal Characteristics.

	12 Month		15 Month			
	WKY	SHR	WKY	SHR		
	(n = 8)	(n = 8)	(n = 8)	(n = 8)	P value†	P value‡
**Body wt (g)**	410±15	420±13	420±20	428±13	0.1	0.2
**Systolic BP (mmHg)**	128±16	191±32	129±10	197±17	<0.0001	0.6
*Echocardiography*						
** LV Fractional shortening (%)**	49.9±3.5	50.6±5.6	51.4±3.6	53.4±3.3	0.5	0.3
** IVS (mm)**	1.4±0.4	2.1±0.2	1.5±0.1	2.2±0.2	<0.0001	0.3
** RA area (mm2)**	14.8±1.1	15.8±1.9	14.1±1.8	16.3±1.2	0.03	0.9
** LA area (mm2)**	19.1±2.0	23.4±2.8	19.0±4.2	23.4±0.6	0.002	0.9
*Pathology*						
** Heart wt (g)**	1.08±0.11	1.34±0.12	1.11±0.07	1.46±0.06	<0.0001	0.03
** RA/body wt (mg/g)**	0.043±0.009	0.051±0.007	0.042±0.013	0.069±0.008	0.0001	0.009
** LA/body wt (mg/g)**	0.049±0.011	0.051±0.006	0.055±0.023	0.085±0.021	0.02	0.004
** IVS/body wt (mg/g)**	0.57±0.04	0.73±0.05	0.58±0.14	0.75±0.12	0.0002	0.8
** RV free wall/body wt (mg/g)**	0.46±0.07	0.50±0.02	0.47±0.10	0.56±0.05	0.02	0.3
** LV free wall/body wt (mg/g)**	1.54±0.22	1.83±0.10	1.57±0.17	1.90±0.13	<0.0001	0.4

Wt – weight; BP – blood pressure; LV – left ventricular; IVS – inter-ventricular septum; RA – right atrium; LA – left atrium; RV – right ventricular.

† P value for effect due to hypertension.

‡ P value for effect due to age.

### Anatomical Remodeling

Significant bi-atrial hypertrophy was evident in the SHR as assessed by echocardiography and pathological examinations (Table 1). These atrial changes due to hypertension were accompanied by increased inter-ventricular septal thickness and bi-ventricular hypertrophy while left ventricular systolic function was preserved (Table 1). However, the effect of ageing on atrial anatomical remodeling was non-significant except when atrial weight was indexed to body weight.

### Electrophysiological Remodeling

#### Atrial refractoriness

Atrial ERP was significantly lower in the SHR as compared to the WKY controls at both time points (p = 0.008). However, in both SHR and WKY, ERP was not significantly different between the 12 and 15 month atria (p = 0.3) and ERP was unaltered by pacing cycle length in both groups (p = 0.8). To illustrate the above, ERP data from the LAFW are presented in Figure 1 (top row). However, when analyzed according to pacing location, ERP was found to be consistently higher in the LA as compared to the RA (Figure 1, bottom row: data from S1 @200 ms; p<0.001). Nevertheless, overall mean ERP heterogeneity was similar between SHR and WKY (LA: 32±8 vs. 31±7%, p = 0.8; RA: 17±9 vs. 23±2%, p = 0.2 respectively).

#### Atrial conduction

**Figure 2:**
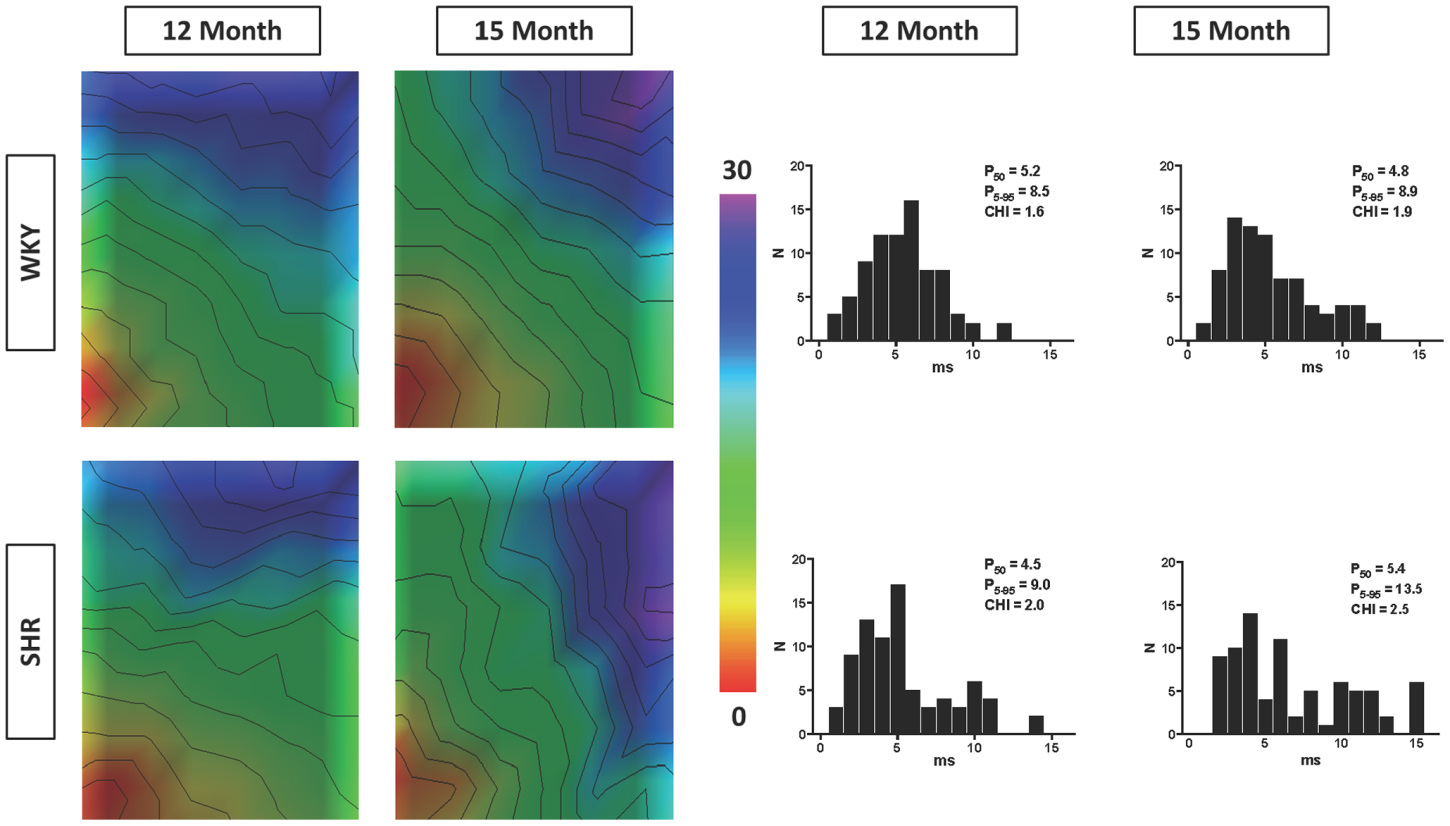
Representative Atrial Activation Maps. Activation maps from representative left atrium of both WKY (top row) and SHR (bottom row) during pacing with S1 at 200 ms are shown here. Isochronal lines of 2 ms are superimposed with fixed time range from 0 to 30 ms. Overall plaque activation times are similar indicating comparable atrial conduction velocity. However, the SHR demonstrated higher conduction heterogeneity as compared to the WKY, which were further increased with ageing. Corresponding phase histograms of these activation maps are also shown on the right panel of this figure.

Figure 2 depicts the activation maps and their corresponding phase histograms from representative LA of WKY and SHR during pacing with S1 at 200 ms. In these maps, isochronal lines are 2 ms apart with early to late activation denoted by the color red to purple. Note that overall plaque activation times are similar indicating comparable atrial conduction velocity. However, the SHR demonstrated higher conduction heterogeneity as compared to the WKY, which were further increased with ageing.

**Figure 3:**
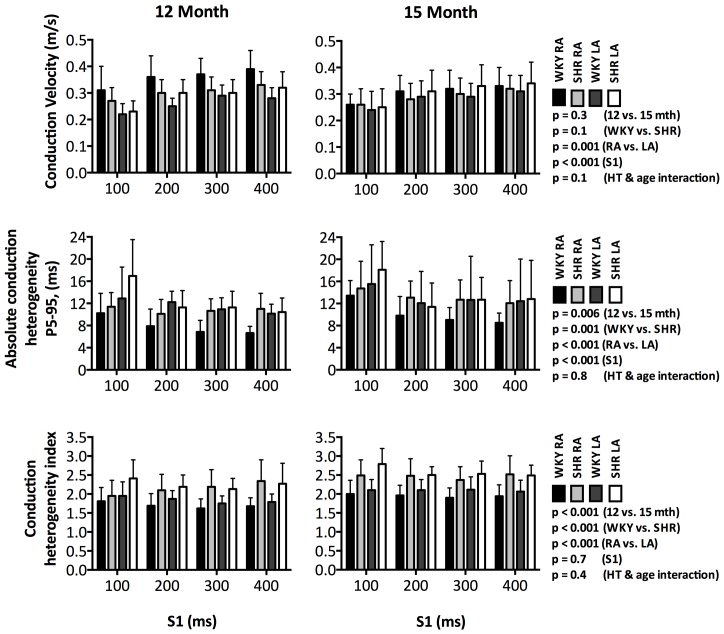
Atrial Conduction. Neither hypertension nor ageing had any significant effect on atrial conduction velocity (top row). In contrast, the SHR atria demonstrated higher absolute conduction heterogeneity (P5–95) and conduction heterogeneity index (P5–95/P50) than the WKY controls (middle & bottom rows). Both P5–95 and P5–95/P50 also increased significantly in tandem with ageing. In addition, increased pacing rate was associated with slower conduction velocity and higher P5–95 (top & middle rows). Significant differences were seen in all three conduction parameters between the left and right atrium.

Indeed, neither hypertension nor ageing had any significant effect on atrial conduction velocity (Figure 3, top row; p = 0.1 and 0.3 respectively). In contrast, the SHR atria demonstrated higher absolute conduction heterogeneity (P5–95) and conduction heterogeneity index (P5–95/P50) than the WKY controls (Figure 3, middle & bottom rows; p = 0.001 & p<0.001 respectively). Furthermore, both P5–95 and P5–95/P50 also increased significantly in tandem with ageing (Figure 3, middle & bottom rows; p = 0.006 & p<0.001 respectively). However, the impact of ageing on conduction velocity, P5–95 and P5–95/P50 was similar in both the SHR and WKY atria as evidenced by the non-significant interaction between hypertension and age for all three parameters (Figure 3, p = 0.1, 0.8 & 0.4 respectively).

Overall, as compared to the RA, the LA demonstrated significantly slower conduction velocity, higher P5–95 and P5–95/P50 (Figure 3: p<0.001, p = 0.001 & p<0.001 respectively). Although atrial conduction was found to be progressively slower with higher P5–95 with increasing pacing rate in both WKY and SHR atria (Figure 3, top & middle rows; both p<0.001), P5–95/P50 was not affected by pacing rate (Figure 3, bottom row; p = 0.3). Nevertheless, all parameters of atrial conduction were found to be direction dependent with higher conduction velocity and lower conduction heterogeneity during cranial-caudal than caudal-cranial propagation [Conduction velocity: SHR - 0.34±0.06 vs. 0.26±0.04 m/s, WKY - 0.35±0.07 vs. 0.27±0.05 m/s; p<0.001, P5–95: SHR - 12.1±7.0 vs. 12.7±4.7 ms/mm, WKY - 9.2±3.6 vs. 11.2±4.6 ms/mm; p = 0.039, P5–95/P50: SHR - 2.20±0.41 vs. 2.52±0.43, WKY –1.89±0.32 vs. 1.93±0.31, p<0.001; respectively].

#### Atrial arrhythmia

**Figure 4:**
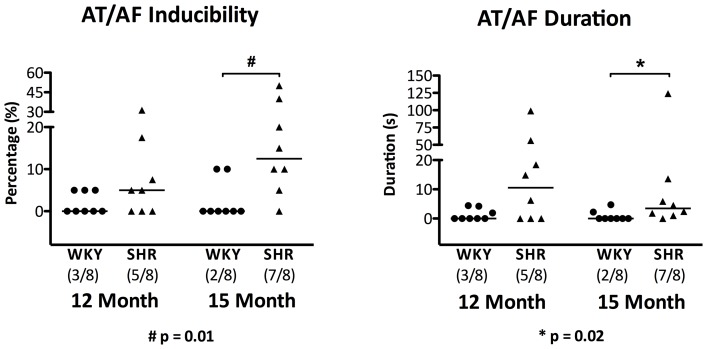
Atrial Arrhythmia Inducibility and Duration. Hypertension resulted in greater arrhythmia inducibility (left panel) and more sustained induced AT/AF episodes in the SHR as compared to normotensive WKY controls at 15 months (right panel). Horizontal lines denote the median values in the scatter plot with the proportion of animals with inducible arrhythmia shown in brackets. However, ageing had no significant effect on both parameters in both groups.

The proportion of animals with inducible atrial arrhythmia following rapid decremental pacing was higher in the SHR than WKY although this was only statistically significant in the 15-month cohort (Figure 4, 12 month: 5/8 vs. 3/8, p = 0.6; 15 month: 7/8 vs. 2/8, p = 0.04 respectively). Likewise, arrhythmia inducibility was significantly higher in the 15-month hypertensive atria as compared to WKY controls (Figure 4, left panel). In addition, induced AT/AF episodes were significantly more sustained in the SHR than the WKY in the 15 months old age group (Figure 4, right panel). However, ageing had no significant effect on both arrhythmia inducibility and duration in both groups as demonstrated in Figure 4. Both macro-reentrant atrial tachycardia and AF were seen in the SHR atria. (Online supplement: Movie S1 and S2 respectively).

### Structural Remodeling

**Figure 5:**
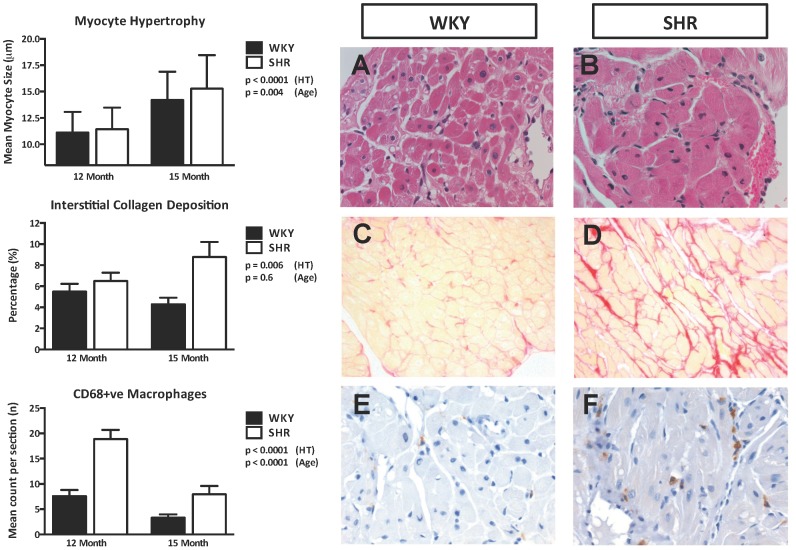
Atrial Structural Changes. Increased atrial myocyte dimension (Left, top panel), collagen deposition (Left, middle panel) and CD68-positive macrophages (Left, bottom panel) were seen with hypertension. Although ageing had no effect on atrial fibrosis, atrial myocyte hypertrophy was evident with lower CD68-positive macrophages count in both SHR and WKY with ageing. Representative H&E (Right, A & B, magnification×400), picrosirius red (Right, C & D, magnification×200) and CD68 immuno-stained sections (Right, E & F, magnification×200) are shown to demonstrate the atrial myocyte hypertrophy, increased amount of interstitial collagen deposition and CD68-positive macrophages in the 15 month-old SHR vs. WKY atria.

The SHR atria demonstrated myocyte hypertrophy (Figure 5, top left panel), increased interstitial fibrosis (Figure 5, middle left panel) and CD68-positive macrophages count (Figure 5, bottom left panel) as compared to the WKY controls. Although ageing had no effect on atrial fibrosis, atrial myocyte size was significantly larger and CD68-positive macrophages count was significantly lower in both SHR and WKY with ageing. Representative photomicrographs of H&E, picrosirius red and CD68 immuno-stained sections from 15 month-old SHR and WKY atria are also shown in Figure 5 (right panel).

## Discussion

This high density mapping study presents detailed characterization of the atrial electrical and structural substrate in mature SHR with established hypertension and left ventricular hypertrophy in comparison to WKY controls, to provide new insights into the relative contribution of hypertension and ageing towards an arrhythmogenic substrate for AF. In comparison to normotensive WKY controls, hypertension in the SHR led to significant: i) Bi-atrial enlargement and bi-ventricular hypertrophy; ii) Abbreviation of atrial refractoriness; iii) Increase in atrial conduction heterogeneity & iv) Increase in atrial myocyte dimension, interstitial fibrosis and CD68-positive macrophages infiltration. Together, these changes resulted in higher atrial arrhythmia inducibility and longer induced AT/AF episodes. In the presence of persistent hypertension and maximal left ventricular hypertrophy, ageing contributed to incremental bi-atrial enlargement, atrial myocyte hypertrophy and atrial conduction heterogeneity without affecting atrial refractoriness. Surprisingly, atrial arrhythmia susceptibility/sustainability and fibrosis were not higher due to ageing in these SHR despite the additional atrial enlargement and conduction changes. Perhaps, the atrial remodeling attributable to ageing was ameliorated by a significant reduction in CD68-positive macrophages. Here, hypertension appears to play a greater pathophysiological role than ageing in the remodeling of the mature SHR atria.

### Concomitant Conditions and Abnormal Atrial Substrate

It is now recognized that co-existing risk factors contribute to both the development and progressive nature of AF. [17], [18] Specifically, ageing and hypertension have also been found to lead to increased thromboembolic complications in patients with AF, even though the pathophysiological contribution or interaction of each factor remains poorly understood. [18] Individually, various animal and human studies on the effect of ageing or hypertension have demonstrated comparable atrial electrical and structural remodeling. The atrial substrate due to ageing consists of unchanged [19] or increased refractoriness or action potential duration[20]–[25] with unchanged/increased dispersion, [22], [24], [26], [27] increased electrogram fractionation, [26], [28] increased delayed afterdepolarizations [24] and decreased conduction velocity. [19], [21], [25], [26], [28] In the hypertensive atria, ERP was found to be unchanged, [5], [6], [29] increased,[30]–[32] or decreased [7], [33] with unchanged [30], [31] ERP dispersion. Other hypertension-related atrial electrical changes included conduction slowing with increased heterogeneity,[29]–[32] increased electrogram fractionation [30], [32] and increased dominant frequency. [33] However, in a mouse model of salt-induced hypertension, conduction velocity was not altered by hypertensive remodeling as seen in this study. [33].

On the structural level, both ageing and hypertension were associated with increased atrial fibrosis and myocyte hypertrophy that could account for the electrical changes of slowed atrial conduction, increased conduction heterogeneity and electrogram fractionation.[5], [6], [19], [21], [29]–[31], [33] The variability in atrial refractoriness could be explained by the differences in species or models studied and the measurement technique (in-vivo vs. in-vitro), but the findings of increased atrial fibrosis and subsequent atrial conduction changes appear to be universal as seen in other arrhythmogenic substrates.[34]–[43] In the present study, we identified similar electrical and structural changes in the mature SHR atria. In addition, we were able to delineate the relative contribution of hypertension and ageing to the abnormal atrial substrate and demonstrate the incremental changes when the two conditions co-exist.

### Pathophysiological Role of Inflammatory Macrophages in Atrial Remodeling

The universal morphological finding of increased atrial fibrosis is likely to represent a final common pathway of structural remodeling that contributes to the maintenance of AF. Indeed, several pathophysiological signaling pathways are implicated in different structural heart diseases leading to the common manifestations of hypertrophy and fibrosis. [3] Recent pre-clinical studies have highlighted an inflammatory role in hypertensive atrial remodeling that had not been well appreciated previously. In one-kidney, one-clip large animal model, we have demonstrated increased inflammatory cell infiltration in short term hypertension and its significant correlation with atrial conduction abnormalities and AF inducibility. [30], [31] Kume and co-workers confirmed the involvement of inflammatory pro-fibrotic mechanisms in a small animal pressure overload model of hypertension due to aortic constriction. [44] These findings corroborate well with clinical data whereby higher C-reactive protein levels were seen in hypertensive patients and associated with higher AF burden and increased risk of developing the arrhythmia.[45]–[47].

Furthermore, active inflammation and immune response in human fibrillating atria have been reported with recruitment of CD68-positive macrophages found across the atrial endocardium. [48], [49] Similarly, we found increased CD-68 positive macrophages in the mature SHR atria. These macrophages are likely initiators of atrial fibrosis through generation of reactive oxygen species, release of cytokines, growth factors and pro-fibrotic enzymes. [50] In addition, the significant ageing related reduction in CD-68 positive macrophages in our study is in keeping with human data whereby both the decline in macrophage numbers and function are well documented with ageing. [51] Taken together, while this could explain the relatively limited incremental atrial remodeling due to ageing in the SHR atria, it further highlights the important pathophysiological role of inflammation in the hypertensive atria.

### Clinical Implications

Hypertension accounts for more AF than any other risk factors due to its high prevalence in the population. As the prevalence of hypertension also increases with age, its arrhythmic burden is likely to be higher in the elderly. This study unravels the relative greater contribution of high blood pressure than ageing to the abnormal arrhythmogenic substrate in the hypertensive atria with established left ventricular hypertrophy. Our findings raise the importance of strict blood pressure management in the elderly, especially those with concomitant AF. This study also presents evidence regarding the important pathophysiological role of inflammatory macrophages in hypertensive atrial remodeling. Novel agents targeting these macrophages derived inflammatory pathways may provide alternate substrate specific upstream therapies in the future.

### Study Limitations

The SHR is the most widely studied experimental model of hypertension. However, it remains uncertain whether we can directly extrapolate our findings to the wide range of human hypertensive syndromes. To facilitate high-density contact mapping of the atria, electrophysiological evaluations were performed ex-vivo in the superfused atria with 2,3-butanedione monoxime added to suppress motion artifacts. Although 2,3-butanedione monoxime has been reported to affect a number of cardiac ion channels, intracellular calcium levels and action potential duration, the dosage used in this study was significantly lower (order of 10×) than those used in the previous reports. We did not verify that the degree of oxygenation and substrate delivery were sufficient to meet the metabolic demands of the atrial tissues. Placement of a lightweight mesh over the atrial tissue on the MEA may induce mechanical stretch and affect atrial electrophysiology. However, at no time did we observe any degradation of signals with our MEA setup, with good tissue viability and high quality of electrogram recordings maintained well after completion of electrophysiological assessment. [9] Data from ex-vivo mapping may be different from in-vivo studies. Nevertheless, we controlled for these limitations by comparing our electrophysiologic data to control atria studied in the same preparations. A bigger gap in the age of the animals would have provided a better picture on the remodeling due to ageing. Diastolic dysfunction was not assessed but previous studies have clearly demonstrated increased diastolic stiffness in the SHR leading to increased atrial stretch [11], [52] that may have contributed to the remodeling seen in the ageing SHR atria described in this study.

## Conclusions

Significant atrial electrical and structural remodeling was seen with hypertension and ageing in the mature spontaneously hypertensive rat atria. In this study, hypertension appears to play a greater pathophysiological role than ageing. Inflammation is likely to be mechanistically important in the pro-fibrotic cascade of the hypertensive atria.

## Supporting Information

Methods S1 **Supplement Methods.**
(DOC)

Movie S1 **This normalized voltage movie demonstrates a stable counter-clockwise wavefront propagation sequence consistent with a macro-reentrant type atrial tachycardia in the left atrium of a 12 month old spontaneously hypertensive rat.** Here, the signal scale ranged from red to purple, representing normalized voltage from 0 to 1.
(WMV)

Movie S2 **This normalized voltage movie shows irregularly irregular atrial wavefront propagations consistent with atrial fibrillation following delivery of a premature atrial stimulus in the left atrium of a 15 month old spontaneously hypertensive rat.** Similarly, the signal scale ranged from red to purple, representing normalized voltage from 0 to 1.
(WMV)
