# Supplementary material for: Atrial Arrhythmia in Ageing Spontaneously Hypertensive Rats: Unraveling the Substrate in Hypertension and Ageing
Source: PLoS One. 2013 Aug 27;8(8):e72416. doi: 10.1371/journal.pone.0072416 (PMC3754972; doi:10.1371/journal.pone.0072416)
Supplement: Methods S1 — Supplement Methods. (DOC) [file pone.0072416.s001.doc]

**Online Supplement Methods S1:**

**Methods:**

In order to visualize and assess activation pattern, a normalized voltage algorithm was implemented to screen the wavefront propagation sequence. The normalized voltage was calculated in two steps:

*1. Calculation of a measure of the local variability of the signal using sliding window:*


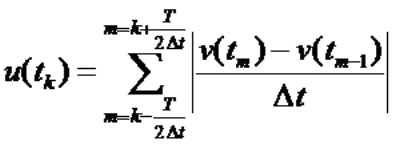


where *T* is the size of the sliding window (20 ms in this study), and *v(t)* is original voltage signal at time *t*. Function *u(t)* increases in value when sliding window starts to overlap with deflection in signal and has a low value in periods of electrical silence.

*2. Normalization of u(t) according to peak value within predefined segments*

Whole signal was then divided into segments of 200 ms length. Within each segment, *u(t)* was divided by a maximum value of *u(t)* within this segment. Therefore, the resulting signal (referred to as the normalized voltage) ranges between 0 and 1.
